# Relief of Ingrowing Toe-Nail

**Published:** 1894-07

**Authors:** 


RELIEF OF INGROWING TOE-NAIL.—Dr. Roswell Park (*Medical News*) quotes Dr. Bogman, of Zanesville, O., as stating that since 1872 he has operated twenty-three times with satisfaction to his patients and himself, adding:

"Such nails are usually thickened or very brittle. I, therefore, soak the foot in warm water to soften the nail and then place it in cold water to contract the blood-vessels. With a strong, pointed knife, I cut, not split, the nail longitudinally along the line of first indication of incurvation, from before backward, and carry the incision backward to and *beyond* the matrix. With another thin knife I deepen the incision vertically to two-thirds the thickness of the toe, keeping close to the bone, but avoiding the periosteum. I then incise the flesh on the same side of the toe parallel to the incision in the nail, forming a flap of the integument and as much as may be of the cellular tissue, thus forming anteriorly, a V. Grasping the portion to be excised entirely with the forceps and lifting it to near the matrix, I dissect out as thoroughly as possible its full width and depth, removing the matrix intact. Closing the wound with adhesive strips, there is present a hair line of cicatrix along the side of the nail, but opposite the matrix is a small point which must heal by granulation. The worse deformed case on which I operated was a patient, a druggist without a clerk, driven to immediate operation by extreme suffering, who was detained from his business but two and a half hours, and this principally by nausea from the anesthetic. Both toes were operated on, on both sides, and all dressings were removed on the eighth day without inconvenience. I gain by this form of operation the smallest possible amount of cicatrix, the preservation of an adequate portion of the nail and the entire ball of the toe, with least detention from business.—*Ex.*

